# Resection of the Elbow Joint and Ulna; Recovery, with a Useful Member

**Published:** 1858-12

**Authors:** J. W. Freer

**Affiliations:** Prof. of Anatomy in Rush Medical College, Etc., Etc.


### RESECTION OF THE ELBOW JOINT AND ULNA. RECOVERY, WITH A USEFUL MEMBER.

BY J. W. FREER, M.D., PROF. OF ANATOMY IN RUSH MEDICAL COLLEGE, ETC., ETC.

*History.*—Zachariah B., aged 17 years, came under my observation, for the first time, in October, 1857, with extensive disease of the elbow joint and ulna of the right side. His medical attendant informed me that six months prior to this, the patient was seized with what appeared like inflammatory rheumatism in the affected joint. At the expiration of five or six weeks he became aware of the true nature of the case by the appearance of an abscess communicating with the articulation. On exploring which, it was found that the articular cartilages were already partially destroyed by ulceration, and the contiguous bones softened and carious. Subsequently the entire ulna became involved in inflammation, eventually leading to necrosis.

At the time specified above, when the patient first came under my care, his general condition was as follows: emaciation, diarrhœa, hectic fever, night sweats, loss of appetite, etc.

*Condition of the limb.*—The arm in semi-flexed position, and incapable of movement; elbow joint much enlarged; on the ulnar side of the joint three sinuses, leading to the articular cavity, one conveniently situated for exploration of the joint, through which it was ascertained that the articular cartilages were nearly, if not altogether, destroyed; the contiguous bones softened and carious; two or three inches below the joint a sinus, leading to the necrosed ulna; at the lower extremity of the ulna a large opening, through which the head of the bone protruded, it having been severed from its ligamentous and articular attachments by the progress of the malady. The radius seemed unaffected by the disease.

In consultation with my friends, Drs. Hollister, Hoffman and Nelson, an operation for resection of the diseased parts was considered advisable, which was performed in the following manner:

*Operation.*—The patient having been placed fully under the influence of ether, an incision was made the whole length of the inner border of the ulna, and extending three inches above the elbow joint. Another at right angle with the former, extending from one condyle to the other. The flaps included in these incisions being dissected up, the posterior surfaces of the joint and ulna were freely exposed; next the triceps muscle and the ligamentous connections of the joint were severed, liberating and bringing into view the diseased surfaces of the articulation. (Special care was taken to avoid injury of the ulna nerve.) Then flexing the forearm, in order to render the humerus prominent, the dissection was carried up the anterior surface to the required extent, and the diseased portion removed with the saw, care being taken to preserve the soft tissues by the interposition of a piece of pasteboard. The point selected for dividing the bone was situated half an inch above the upper edge of the olecrenal fossa.

Then, proceeding to the inferior constituents of the joint, the head of the radius was removed by means of the bone scissors; after which, the ulna was enucleated entire, great care being observed to preserve the periosteum with its patches of new bone developed irregularly here and there. Two or three small vessels required ligation. The incisions were closed with interrupted suture; the limb placed in a flexed position, secured by an angular splint of pasteboard; a full dose of morphine ordered, and the patient placed in bed.

*Morbid anatomy of the parts.*—The articular surface of the humerus completely denuded of cartilage, with considerable destruction of the corresponding bone; the cartilaginous surface of the head of the radius in a state of progressive ulceration; the sigmoid notch of the ulna denuded of cartilage; the bone in a carious condition; the shaft of the ulna necrosed throughout its length, which was previously involved in irregular patches of provisional bone, not sufficiently firm to offer any obstacle to the removal of the sequestrum.

The patient remained under the care of the attending physician of the almshouse (Dr. Nelson), who informed me that he recovered rapidly and without reverses.

At the present time, nine months after the operation, the limb is in the following condition: the elbow firmly anchylosed; the original situation of the ulna nearly occupied with new bone; the hand and fingers useful, and capable of performing all of the natural movements, but with less vigor than is desirable, owing, undoubtedly, to the detachment of the origin of the long flexors and extensors, and the long period of disuse of the member.

*Remarks.*—The propriety of resection of the elbow joint, in suitable cases, is so well established that its utility is unquestionable. Happily for mankind conservative surgery is "the order of the day." But until recently, the practicability of removing the elbow joint, with one or the other of the bones of the forearm, has been doubted, at least, with any prospect of restoring the member to usefulness.

In refutation of this doubt, several illustrative cases have been recently presented to the profession. Two by Prof. Carnochan, wherein in one case he removed the ulna, in the other the radius, both resulting in useful members. Another by Prof. Brainard, in which the ulna was removed with the constituents of the elbow joint, resulting in an unusual degree of usefulness of the member. And it gives me much pleasure that I am able to add still another case in support of the utility and practicability of this truly conservative operation.

The present tendency of the professional mind is such, that at no remote period, resection of a certain class of diseased joints will probably completely substitute the original destructive method by amputation. We have already gained sufficient experience to establish the comparative safety in the removal of even the large articulations, such as the knee, hip joint, etc.; and it is desirable that these inquiries should be extended, until the operation, as well as the diseases amenable to its benefits, are fully systematized.

Professors Davis and Byford,—It is due to *you*, as publishers of the "Journal," to say that, although not at this time a subscriber to your excellent publication, I am a reader of it. My friend, Dr. A. W. Adair, and I take three partnership journals—I receiving the *American* and *Medical News*, and he yours—and we exchange. On motion of Dr. Green, one of the members of the graduating class last winter, it was unanimously voted that I should furnish you with the accompanying paper for publication. This duty I now comply with.

Respectfully yours, etc.,
WM. MATTHEWS.

*Sept.*, 1858.

ARTICLE IV.

